# How to Open the Treasure Chest? Optimising DNA Extraction from Herbarium Specimens

**DOI:** 10.1371/journal.pone.0043808

**Published:** 2012-08-28

**Authors:** Tiina Särkinen, Martijn Staats, James E. Richardson, Robyn S. Cowan, Freek T. Bakker

**Affiliations:** 1 Royal Botanic Garden Edinburgh, Inverleith Row, Edinburgh, United Kingdom; 2 Natural History Museum, Cromwell Road, London, United Kingdom; 3 Biosystematics Group, Wageningen University, Wageningen, The Netherlands; 4 Universidad de Los Andes, Apartado Aéreo, Bogotá, Colombia; 5 Royal Botanic Gardens, Kew, Richmond, Surrey, United Kingdom; University of Florence, Italy

## Abstract

Herbarium collections are potentially an enormous resource for DNA studies, but the use of herbarium specimens in molecular studies has thus far been slowed down by difficulty in obtaining amplifiable DNA. Here we compare a set of commercially available DNA extraction protocols and their performance in terms of DNA purity and yield, and PCR amplification success as measured by using three differentially sized markers, the *rbcL* barcoding marker (cpDNA), the *LEAFY* exon 3 (nrDNA), and the *trnL* ^(UAA)^ P6 loop (cpDNA). Results reveal large differences between extraction methods, where DNA purity rather than yield is shown to be strongly correlated with PCR success. Amplicon size shows similarly strong correlation with PCR success, with the shortest fragment showing the highest success rate (78%, P6 loop, 10–143 base pairs (bp)) and the largest fragment the lowest success (10%, *rbcL*, 670 bp). The effect of specimen preparation method on PCR success was also tested. Results show that drying method strongly affects PCR success, especially the availability of fragments longer than 250 bp, where longer fragments are more available for PCR amplification in air dried material compared to alcohol dried specimens. Results from our study indicate that projects relying on poor-quality starting material such as herbarium or scat samples should focus on extracting pure DNA and aim to amplify short target regions (<200–300 bp) in order to maximise outcomes. Development of shorter barcoding regions, or mini-barcodes within existing ones should be of high importance as only a few options are currently available; this is particularly important if we hope to incorporate the millions of herbarium samples available into barcoding initiatives and other molecular studies.

## Introduction

Herbaria, once called dry gardens, are collections of preserved plant specimens used for scientific study. The earliest herbaria were established in Europe in the 16^th^ century [1], [2], and scientists have since continued to collect from all corners of the globe. It has been estimated that the world’s 2721 active herbaria together house c. 361 million specimens [3]. These collections cover most of the world’s plant species, including many narrowly restricted local endemics, species remaining to be described [4], [5], and those already extinct.

Despite the large number of specimens housed in herbaria world wide, currently only a small fraction is being used for DNA-based research, mainly due to poor success of extraction and PCR amplification of most herbarium DNA. Hence, systematic studies are needed to optimise methods and their efficiency. This is particularly relevant at times when it is far easier and cost effective to obtain herbarium specimens from diverse geographic localities than living material, particularly when species are becoming extinct or increasingly rare in the wild. Making herbarium DNA more accessible would also contribute to ongoing plant barcoding initiatives, such as iBOL (iBOL.org). iBOL aims at providing a DNA-based identification tool for majority of plant species by 2015, and 10% of the targeted DNA barcodes are planned to be taken from museum material. Access to herbarium DNA would therefore help such projects to sample species diversity much more efficiently, as herbaria are the largest access points to expert verified plant samples.

### Herbarium DNA and its Challenges

**Figure 1:**
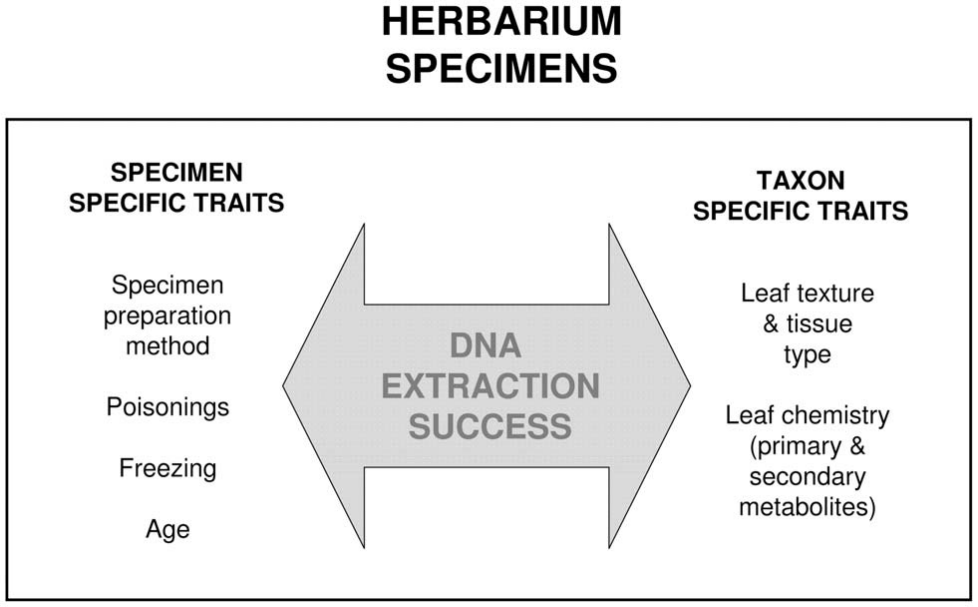
Factors affecting success of DNA extraction from herbarium specimens.

Herbarium DNA presents particular challenges to molecular studies that can be broadly divided into (1) specimen specific, and (2) taxon specific factors (Figure 1). The specimen specific issues include factors such as sample preparation method, preservation history, and specimen age. Previous studies have demonstrated that most damage in herbarium DNA is caused by specimen preparation, with varying degrees of damage caused by different drying methods used, with only a marginal effect of subsequent preservation history [6]–[8], and no detectable effect of specimen age [9]. Staats et al. [6] also showed that there is no bias in the preservation of nuclear versus organelle DNA, indicating that nuclear and commonly used plastid markers are available for PCR amplification in the same ratio as in fresh tissue.

Taxon specific factors of herbarium DNA include the outstanding diversity of leaf textures and the wide array of secondary compounds present in plants (Figure 1). Plants are much more diverse in their chemistry compared to other organisms, and of the thousands of primary and secondary metabolites found in plants, polyphenols and polysaccharides affect DNA extraction most severely through DNA oxidation, covalently binding to nucleotides and by inhibiting enzymatic reactions during PCR [10]–[12]. Particular leaf types and textures can similarly hinder DNA extraction. Known or potentially problematic groups include succulents (e.g., Crassulaceae, Aloeaceae, Cactaceae) [13], [14], hard- and fibrous-leaved species (e.g., Aquifoliaceae, palms) [15], carnivorous plants [16], and taxa with resin or sap (e.g., Apocynaceae, Pinaceae, Sapotaceae) [17].

Despite previous studies conducted on specific aspects of herbarium DNA (e.g. [18]–[21]), no large scale studies have been done on testing the effect of different DNA extraction methods on DNA extraction efficiency from herbarium specimens across a broad range of taxa. Although innovative methods of DNA extraction and PCR amplification are being developed that function well with low quantity DNA (e.g., [22]–[24]), few are currently available for large-scale projects aimed at working across plant taxa using automated or semi-automated protocols. The available methods include (1) silica binding, (2) magnetic bead binding, (3) salting-out precipitation, and (4) anion exchange purification. Each is based on a different DNA extraction technique and chemistry, and hence the methods are expected to vary in their DNA extraction efficiencies.

Here we provide a systematic study of DNA extraction efficiency from historic herbarium specimens representing a broad range of phylogenetic diversity of vascular plant species and preservation histories. We test eight commercially available protocols and protocols that are commonly used in laboratories specialised in processing herbarium samples, representing each of the above-mentioned four DNA extraction methods. We also test the effect of preservation method on DNA extraction efficiency through experimentally drying specimens using silica drying, natural air-drying, artificial air-drying, and alcohol drying (both quick and slow). General conclusions are drawn on the relative performance of different extraction methods, DNA polymerases, PCR additives, using three different molecular markers, and recommendations for future studies are given.

## Results

### DNA Polymerase-specific Effect on Amplification Success

Four DNA polymerase enzymes were tested in order to select the best performing enzyme for further experiments. Results showed large differences between enzymes in terms of amplification success, which we define qualitatively here as the ability to amplify the barcoding region *rbcL*. Platinum Taq was the best performing polymerase with the highest number of positive amplicons for the tested marker (*rbcL*, 19% success rate) (Table 1). The 3′–5′ proofreading enzymes Platinum Taq High Fidelity and SAHARA both performed poorly with only 11% and 2% *rbcL* PCR success rate, respectively (Table 1). The effect of the repair enzyme PreCR Repair Mix was tested with the best performing DNA polymerase Platinum Taq, and showed that whilst no additional positive amplicons were seen (Table 1), the repair enzyme produced higher yielding amplicons (results not shown).

**Table 1:** Performance of different DNA polymerase enzymes tested.

	DNA polymerase enzyme	No of positive amplicons (*rbcL*)	PCR success rate (%)
**1.**	BioTaq	9	19
**2.**	SAHARA	1	2
**3.**	Platinum Taq	12	26
**4.**	Platinum Taq High Fidelity	5	11
**5.**	PreCR Repair Mix + Platinum Taq	12	26

### DNA Yield and Purity

**Figure 2:**
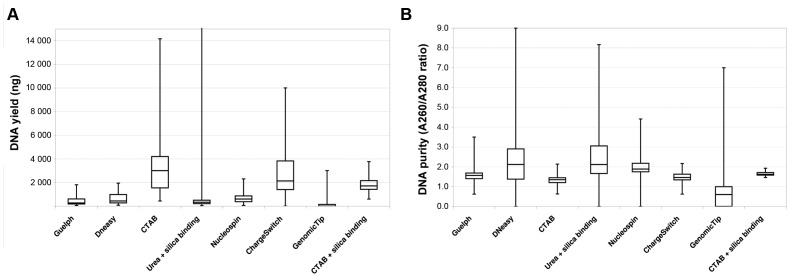
Effect of extraction method on DNA yield and purity.

The eight tested extraction protocols showed statistically significant differences both in terms of DNA yields (Friedman’s Rank test, *P*<0.00001) and DNA purity ratios (*P*<0.00001). Highest median DNA yield was obtained with the CTAB method (3000 ng) followed by the ChargeSwitch protocol (2076 ng), whilst GenomicTip protocol gave the lowest median yield (7 ng) (Table 2) (Figure 2). Protocols yielding high amounts of DNA generally performed poorly in terms of DNA purity showing low A260/A280 ratios (1.36–1.46) (Figure 2), whilst protocols with low DNA yield showed pure DNA (Table 2). The purest DNA was obtained using the NucleoSpin protocol with median purity of 1.88 (40% of samples pure), followed by the CTAB + silica binding approach which gave median purity of 1.62 (22.9% of samples pure) (Table 2).

**Table 2:** Performance of different DNA extraction methods with historic plant herbarium specimens; results of the three best performing methods are in bold under each category.

Protocol used	Extraction method	Reference/supplier (catalogue #)	Median DNA yield (ng)	Median DNA purity (A260/A280)	No of samples with pure DNA (%)^3^	No of positive amplicons (%)			
						*rbcL*	*LEAFY*	P6 loop	Combined
DNeasy Plant Mini Kit	Silica binding	Qiagen (69104)	434	**2.118**	7 (14.5)	**10 (21)**	**16 (34)**	**45 (96)**	**45 (96)**
NucleoSpin Plant Kit II	Silica binding	Machery & Nagel (NZ74077050)	593	**1.884**	**19 (39.6)**	**9 (19)**	**19 (40)**	41 (87)	**42 (89)**
Canadian Centre for DNA barcoding Guelph, Ontario	Silica binding	Ivanova et al. [25]	285	1.564	**8 (16.7)**	2 (4)	14 (30)	35 (74)	29 (62)
CTAB^1,2^+ Wizard DNA Clean-up System (Promega)	Silica binding + Salting-out precipitation	Doyle and Doyle [26] + Promega (A7280)	**1712**	**1.620**	**11 (22.9)**	3 (6)	**31 (66)**	**47 (100)**	**47 (100)**
Urea pre-treatment^2^+ DNeasy Plant Mini Kit	Silica binding + Salting-out precipitation	Qiagen (69104)	330	2.116	4 (8.3)	2 (4)	5 (11)	41 (87)	41 (87)
CTAB^1,2^	Salting-out precipitation	Doyle and Doyle [26]	**3004**	1.350	2 (4.2)	0 (0)	4 (9)	38 (81)	37 (79)
ChargeSwitch gDNA Plant Kit	Magnetic bead binding	Invitrogen (CS18000)	**2076**	1.459	**8 (16.7)**	3 (6)	8 (17)	14 (30)	16 (34)
Genomic Tip 20/G	Anion exchange purification	Qiagen (10223 & 19060)	7	0.602	3 (6.3)	**6 (13)**	12 (26)	**42 (89)**	34 (72)

1 CTAB (cetyltrimethylammonium bromide lysis) with PVP and β-mercaptoethanol.

2 See File S1 for details of extraction protocol used.

3 A260/A280 ratios 1.7–2.0 were considered pure.

### PCR Amplification Success

**Figure 3:**
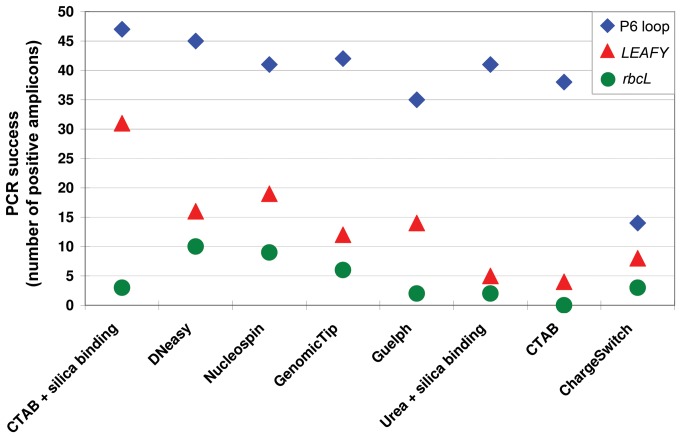
Effect of extraction method on PCR success as measured by number of positive amplicons. RM = room temperature.

All herbarium specimens sampled produced amplifiable DNA with at least one of the DNA extraction protocols tested here. The DNA extraction protocol with the best overall PCR success, i.e. the highest number of herbarium specimens with at least one positive amplicon, was the method combining CTAB with silica binding (100%) followed by the DNeasy protocol (96%) (Table 2, Figure 3). The highest number of *LEAFY* (66%) and P6 loop (100%) amplicons were obtained with CTAB + silica binding, whereas the highest number of the relatively long *rbcL* amplicons (23.4%) was obtained with the DNeasy protocol (Table 2, Figure 3). There was a strong negative trend between amplicon size and PCR success, with the short P6 loop (c. 10–143 bp) amplifying consistently better than the long *rbcL* (c. 670 bp) and median sized *LEAFY* (c. 260 pb) (Table 3), indicating that shorter fragments are easier to amplify from herbarium DNA. There was no statistically significant difference in median DNA yields between DNA extracts with and without PCR success (Mann-Whitney Test, Z = −1.267, *P* = 0.205), indicating that DNA yield is not predictive of PCR success. There was, however, a statistically significant difference in the median DNA purity (A260:A280 ratio) between DNA extracts with and without PCR success (Mann-Whitney Test, Z = −5.120, *P*<0.001), indicating that DNA purity can be used as a predictor of PCR success. DNA extraction protocols which showed high PCR success had a higher number of samples with pure DNA ratios (median = 1.65, range = 1.40–2.07) compared with DNA extraction protocols which showed no PCR success (median = 1.37, range = 1.04–1.62).

**Table 3:** Variation in amplification success between the three sequencing regions used.

Region	Length (bp)	Genome	Universal primers	PCR success	
				No of positive amplicons	Success rate (%)
*rbcL* barcode region	670	plastid	yes	32	10
*LEAFY* exon 3	260	nuclear	yes	78	24
*trnL*(UAA) P6 loop	10–143	plastid	yes	256	78

Although age was not expected to affect PCR success, we tested for signal and found no effect of specimen age on PCR success (Mann-Whitney Test, Z = −1.489, *P* = 0.136). Sequencing of amplicons confirmed that all tested samples had their expected identity based on BLAST searches, indicating that contamination had no effect on our PCR results. In one case (P6 loop sequence of the sample 14 originally determined as *Commelina communis* L., Commelinaceae), BLAST search returned a close match to *Weldenia candida* Schult. f., Commelinaceae (E-value = 1e–16) but this was due to misidentification of the original voucher rather than contamination. The sample has now been re-identified as *Tradescantia* sp. (Table S1).

### Effect of Specimen Drying Method on Herbarium DNA

**Figure 4:**
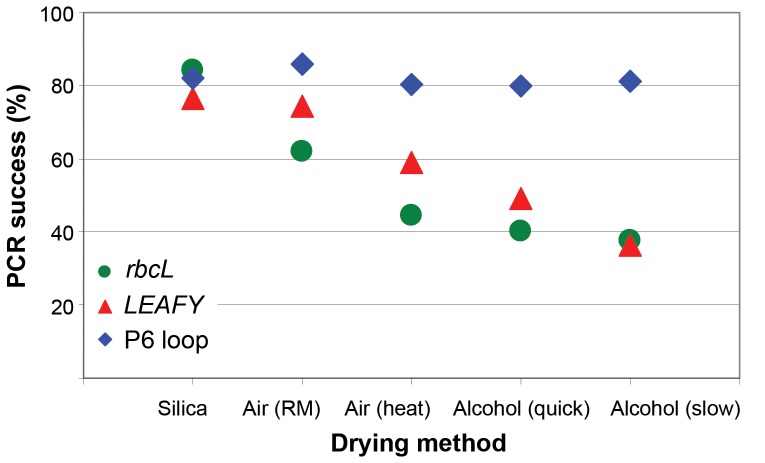
Effect of sample preparation method on PCR success (%), measured as the number of positive amplicons divided by the total number of samples.

The five herbarium specimen drying methods tested showed statistically significant differences in DNA yields (Friedman’s Rank test, *P = *0.004). Alcohol drying (quick and slow) showed lowest median DNA yields (533–652 ng), whilst air drying with artificial heat gave the highest median DNA yield (1322 ng) (Table 4). There was no statistical difference in DNA purity between the five drying methods tested (Friedman’s Rank test, *P = *0.153). PCR success was 100% for all three regions amplified for the silica gel dried leaf material (Table 4, Figure 4). For other methods, success varied depending on the target amplicon size and the drying method used (Table 4, Figure 4). The P6 loop amplified for nearly all samples of all drying methods, but for both *LEAFY* and *rbcL*, PCR success was highest in DNA extracted from air dried material (*rbcL* 78–84%, *LEAFY* 72–89%), and lowest for the alcohol dried specimens (*rbcL* 61%, *LEAFY* 61–72%) (Table 4, Figure 4). The drying method using paper blotting at room temperature had the highest PCR success rate after the silica drying method (Table 4).

**Table 4:** Performance of different methods of specimen preparation in relation to DNA extraction success.

	Method	Duration of drying	Median DNA yield (ng)	Median DNA purity (A260/A280)	PCR success: No of positive amplicons (%)		
					*rbcL*	*LEAFY*	P6 loop
**1.**	Silica	1 day	1312	1.866	18 (100)	18 (100)	18 (100)
**2.**	Air (heat)	12 hours	1322	1.894	14 (77.8)	13 (72.2)	18 (100)
**3.**	Air (room temperature)	2 weeks	967	1.895	15 (83.3)	16 (88.9)	18 (100)
**4.**	Alcohol (quick)	Overnight	652	1.979	11 (61.1)	13 (72.2)	18 (100)
**5.**	Alcohol (slow)	2 weeks	533	1.887	11 (61.1)	11 (61.1)	17 (94.4)

## Discussion

### DNA Purity Before Quantity

Our study is the first to systematically compare different DNA extraction methods on a phylogenetically diverse panel of angiosperm herbarium specimens and highlights the importance of aiming for high DNA purity, rather than quantity in herbarium studies. The importance of high purity, contaminant-free DNA has been acknowledged in ancient DNA studies, and several studies have focused on developing extraction protocols optimising not only yield but purity [27]–[30]. The tight link between DNA purity and PCR success can be expected particularly in plants due to the vast array of primary and secondary chemicals present in their cells. Failure to clean DNA of polyphenols and polysaccharides can result in negative PCR results despite high DNA yields due to the PCR inhibiting properties of primary and secondary chemicals even in non-degraded DNA samples. The importance of high purity DNA can hence be expected to be even higher for degraded plant samples such as herbarium DNA compared to ancient DNA of other organisms. Best performing DNA extraction protocols for herbarium DNA are those that combine high purity with high DNA yield, such as our combination method of CTAB with silica binding. These methods hold much promise and focus should be given to their further development and upscaling.

### Small Fragments in Herbarium DNA

The second factor that strongly affects PCR success is the target amplicon size. Our results show that short fragments are abundant in herbarium DNA and hence PCR of smaller target regions have higher success rates. Short fragments in degraded DNA samples has been flagged as a problem since the beginning of fossil DNA studies (e.g., [31], [32]), and our study is the first to explore the availability of differently-sized fragments across a panel of old herbarium specimens. We used three differentially sized regions to test the effect of target locus size on amplification success, and our results indicate that there is a sharp cut-off point in the availability of fragments around c. 200 bp: PCR success rates were close to 100% for the 100 bp long region, 24% for the 260 bp region, and only 10% for the longest region (670 bp). Aiming for regions shorter than 300 bp would be advisable for projects working on degraded plant samples, but further studies are needed to establish the upper and average size limits of available fragments in herbarium DNA, e.g. following methodology by Zimmermann et al. [32]. The situation will be slightly different, however, in projects that apply next-generation sequencing technologies (see Discussion below).

### Effect of Other Factors on Herbarium DNA PCR Success

Our study also took into consideration other factors such as choice of DNA polymerase enzyme and PCR additives on PCR success. We found large differences in polymerase performance with herbarium DNA. Our results follow recent studies which have demonstrated that the choice of polymerase greatly influences PCR success [33], [34] through not only different enzyme structure and catalytic properties but also due to differences in enzyme purity and polymerase buffer chemistries [33]. Our results further suggest that enzymes with 3′–5′ proofreading capacity perform worse on herbarium DNA compared to enzymes without proofreading capacity in terms of amplification success.

There is now ample evidence that high concentration BSA has a strong positive affect on PCR success on poor quality template DNA. The evidence comes from various studies which have focused on herbarium DNA [19], fossil plants [35], and ancient mammal DNA [34]. Hence, the use of high concentration BSA in PCR reactions should be established as a routine protocol in herbarium DNA studies. Studies on ancient mammal bone tissues have also explored the effect of other additives such as Triton-X 100, Tween 20, proteinase K and N-lauryl-sarcosine on DNA extraction [34], but thus far no systematic study has been performed in plants. Further studies are needed to establish what additives would be cost effective in DNA extraction or PCR to counteract the negative effects of primary and secondary chemicals such as polyphenols and polysaccharides. However, given the extensive phytochemical spectrum across vascular plants, general solutions may not be possible. Whilst our study focused on optimising large-scale DNA extraction from herbarium specimens across a broad range of taxa, various taxon-specific optimal DNA extraction protocols have been published and may be applied when working on a narrower range of species.

### Predicting PCR Success for Specimens

Our results on the effect of sample preparation method on DNA quality confirm the general belief that alcohol treated plant material is not optimal for DNA studies. Specimens dried using alcohol pre-treatment yielded lower amounts and more fragmented DNA compared to air dried specimens. Both short (1 day) and long (2 weeks) alcohol treatment resulted in highly fragmented DNA, suggesting that it is not the duration but the use of alcohol pre-treatment as such that causes DNA damage. Differences were clear when comparing the results from the alcohol dried material with the air-dried material that were treated for the same short time period: alcohol treated material showed significantly lower DNA yields, and lower PCR success with large and medium sized amplicons. No significant differences were observed between the two air-drying methods similar to results by Harris [7], suggesting that natural drying over longer time periods in lower heat is equally good compared to drying specimens quickly in higher temperatures.

Although our results are useful in guiding how collectors might collect their specimens in the field in the future, these results do not help in predicting which herbarium specimens in our existing collections should be used for DNA studies. Details of specimen preparation method are generally not available for specimens, and in most cases, it is impossible to ascertain specimen drying method post-hoc. We blind tested four senior experienced field botanists at the Royal Botanic Garden Edinburgh with a set of 40 specimens, confirming that expert opinion was highly contradictory. Historical facts regarding collection methods can, however, help in elucidating some patterns. Air drying without artificial heat was used exclusively prior to the 1880s by researchers such as Darwin during local and even more global collection trips. In 1887, alcohol drying, also known as the Schweinfurth method, was established as a method for collecting plants in the humid tropics and was advertised widely during the consecutive years [36]–[38]. To date, alcohol drying remains in use predominantly in South East Asia but also in Latin America and Africa in projects working in remote areas. Specimens collected in temperate locations continue to be exclusively air-dried, either naturally or using artificial heat.

### Implications

Results from our study should act as a starting point for future studies on optimization of DNA extraction protocols for herbarium material. Following Telle and Thines [33] and Roland and Hofreiter [30], [34], effects of various additives and steps of DNA extraction could be explored in detail in order to establish an optimal protocol for herbarium specimens which would maximize primarily DNA purity but also yield, whilst reducing any further damage to DNA. Such studies should pay particular attention to reducing the negative effects of polyphenols and polysaccharides through directly inhibiting enzymatic reactions such as PCR but also indirectly through DNA oxidation.

Our results have important implications for DNA barcoding initiatives such as iBOL (www.ibol.org) that rely heavily on access to well-identified plant material. For rare species, i.e. most of our planet’s diversity, herbaria are the only feasible sources of such material. Our results show that special care should be taken, however, when working with herbarium DNA, as it is usually severely fragmented, strongly limiting the ability to PCR amplify longer fragments. In our study, only fragments <300 bp were easily amplifiable from herbarium DNA with any significant success rate. For large scale studies, increasing success rate would be a high priority in order to reduce costs.

Our results are in accordance with results by Zimmermann et al. [32] who showed that only relatively short fragments 20–100 bp were available in c. 50 year old frog and moth museum samples. Sample preparation methods vary greatly between vertebrate, invertebrate and plant sciences, but the study shows a similar trend to our results here. These studies highlight the importance of developing short mini-barcodes, or metabarcodes sensu Taberlet et al. [39], that could be used for poor-quality samples such as herbarium material (see [40] for an example for animal studies). The current full plant barcodes *rbcL* and *matK* are both too long (>650 bp) to be used for poor-quality samples, and there are so far no other candidates amongst additional proposed barcoding regions [41]. The most promising short region is the *trnL* ^(UAA)^ P6 loop marker as used in this study, which is located within the commonly used *trnL-trnF* region, and is already commonly used in projects working on highly fragmented environmental samples such as animal scat or gut samples used for studying diet and resource use [42]–[44] and environmental core samples used for reconstructing past vegetation [45]. Similar to above, primers have been developed for a mini-barcode of *rbcL* suitable for working with degraded DNA [44], but the use of this minibarcode has been limited as the universality of these primers has not been thus far tested.

These results indicate that DNA studies using traditional Sanger sequencing techniques, which rely on prior PCR amplification, may be problematic. The presence of fragmented DNA should, however, be far less of a problem in a next-generation sequencing world as most NGS sequencing approaches require fragmented (and ligated) DNA as starting material (e.g. [46]). The fact that NGS platforms are based on short sequence reads makes them a promising tool for herbarium DNA. There are some caveats, however. Currently, NGS platforms require high amounts of DNA, and DNA yields obtained from herbarium samples may not be enough for most applications. Possibly, single molecule sequencing technology could prove instrumental for damaged DNA [47]. Meanwhile, the field of meta-barcoding is developing approaches suited for environmental DNA samples which include low abundance taxa with low template DNA yields [39]. Such meta-barcoding approaches could be considered for herbarium DNA, as these would be well suited for the generally low yielding herbarium samples.

In summary, results from our study together with evidence from previous investigations on herbarium DNA, strongly suggests that there are five important factors to be considered when working with herbarium DNA (in no particular order): (1) amplification success is higher for shorter target regions due to severe fragmentation of herbarium DNA; (2) DNA purity is more important as a predictor for amplification success than DNA yield, and hence DNA extraction techniques which maximize DNA purity should be used; (3) BSA should be routinely used in PCR reactions in high concentrations when working with herbarium DNA [19], [34]; (4) specimen preparation method strongly affects PCR success through DNA fragmentation, where specimens treated with alcohol have generally more fragmented DNA; hence shorter target amplicon sizes (c. 100 bp) are recommended for alcohol dried specimens in order to maximize success rates; and (5) there is no biased degradation of nuclear DNA and that organelle (plastid and mitochondrial) and nuclear DNA are equally available in herbarium samples compared to fresh tissue (as reported in [6]).

**Table 5:** Regions and primers used for testing DNA quality through PCR amplification.

Region	Primer			
	Name	Direction	Sequence	Reference
*rbcL* (barcoding)	A_f	Forward	ATG TCA CCA CAA ACA GAG ACT AAA GC	Kress and Erickson [48]
	A_r	Reverse	CTT CTG CTA CAA ATA AGA ATC GAT CTC	Kress and Erickson [48]
*trnL*(UAA) P6 loop	g	Forward	GGGCAATCCTGAGCCAA	Taberlet et al. [45]
	h	Reverse	CCATTGAGTCTCTGCACCTATC	Taberlet et al. [45]
*LEAFY* (exon 3)	Lfl-1	Forward	GCGAATTCACIAAYCARGTITTYMGIYAYGC	Frohlich and Meyerowitz [49]
	Lfl-3	Reverse	CGGAYATIAAYAARCCIAARATGMGICAYTA	Frohlich and Meyerowitz [49]

## Materials and Methods

### Herbarium Test Samples

A set of 47 herbarium specimens from the Royal Botanic Garden Edinburgh (RBGE) herbarium (E) were used for all experiments (Table S1). All necessary permissions for the described plant and specimen sampling were obtained from the respective curators, David Harris and Fiona Inches, RBGE. The samples represented 47 species from 47 families and 46 Angiosperm plant orders, with age range of 52–92 years with a median of 64 (Table S1). Various notorious ‘problem’ taxa were included in order to represent a realistic diversity of leaf textures and chemistries (Table S1). Although specimen preparation method data is not available for any of the historic samples, we used their visual appearance and geographic origin to predict drying method. Specimens collected in the UK were all considered air dried, whilst any specimen with clear wet marks or white powdery remains of alcohol were considered alcohol dried. This led us to predict that 72% of samples were likely air dried, and 15% alcohol dried, with 6 specimens alcohol or air dried (Table S1).

### DNA Extraction

DNA extraction protocols were chosen to cover all four main DNA extraction methods as well as most commonly used protocols in some of the largest plant laboratories working on herbarium DNA. For each method, we chose commercially available kits that are commonly used in laboratories specialised in plant samples, and kits which allow extractions of low-abundance DNA samples. Three of the eight protocols chosen were based on the silica binding method, one on magnetic bead binding, one on anion exchange purification, and one on salting-out precipitation (Table 2). In addition, two protocols combining salting out precipitation (CTAB) and silica binding were tested (Table 2). For commercially available kits, manufacturer’s instructions were followed for poor-quality template samples; for published protocols, original publications were followed, and for the CTAB and Urea protocols, extraction protocol details can be found in File S1. Five mg of leaf fragment was weighed and placed in an Eppendorf tube with a pinch of sterilised sand and a metal bead. Samples were then ground in a Mixer Mill (MM300, Qiagen) in pre-cooled (−20°C) adapters for 4×1 minutes at 20 Hz speed. For all protocols, we treated samples with Ribonuclease A (RNase A) to remove RNA before PCR and gel imaging. DNA was eluted in sterile water, or elution buffer provided by kits.

### DNA Polymerase and Repair Enzyme Test

Previous studies have shown that the choice of DNA polymerase enzymes and the use of additives in PCR can affect PCR success especially in poor quality DNA [19], [33], [34]. We tested four different DNA polymerases which differed in cost/unit: BioTaq (Bioline, BIO-21040) represented a basic, commonly used DNA polymerase, Platinum Taq (Invitrogen, 10966-018) and Platinum Taq High Fidelity (11304-011), the latter with 3′–5′ proof-reading capabilities. Finally SAHARA (Bioline, BIO-21090) was chosen as it is advertised to work well with low-template samples and because it possesses 3′–5′ proofreading activity (Table 1). We also tested the effect of the PreCR Repair Mix (New England Biolabs, M0309S) on subsequent PCR success. The enzyme mixture repairs basic DNA damage such as abasic sites, nicks, thymine dimers, deaminated cytosines, oxidized guanine and pyrimidine, which prevent miscoding nucleotides from being incorporated and lead to DNA polymerase stalling. PreCR was tested with the best performing DNA polymerase (Platinum Taq, see Results) using the DNA extracted with the DNeasy kit.

### DNA Quality Measurements

DNA quality was evaluated using three criteria: (1) DNA purity, (2) DNA yield, and (3) PCR amplification success. DNA purity (OD_260/280_ and OD_260/230_ ratios) and yield were measured using a calibrated NanoVue UV-spectrophotometer (GE Healthcare) on high accuracy mode. OD_260/280_ ratios between 1.7 and 2.0 were considered to indicate pure DNA. We also aimed to measure DNA yield and fragmentation through gel imaging and quantification. Five µl of each sample was run on a 1.8% agarose gel with 0.5 ug/ml of ethidium bromide. This approach failed however, as no or little DNA was visible for most samples other than the control due to the generally poor DNA yields (see Results). Increasing the amount of DNA loaded on to the gel was not possible due to the limited sampling permitted from the herbarium material used.

The ultimate test of DNA quality was PCR amplification success, which was used to test the overall quality and quantity of the template DNA, as well as the level of DNA fragmentation. We define amplification success qualitatively as the generation of specific fragment bands visible on agarose gels. Visual threshold was measured using GeneTools software (Syngene UK) which detects bands on gel images based on expected size. Three markers were used, two chloroplast markers and a single copy nuclear gene marker (Table 5), all ranging in size between 10–143 (P6 loop), 260 (*LEAFY*), and 670 bp (*rbcL*). We chose *rbcL* and the P6 loop as these represented two different length chloroplast markers and there are universal primers available for both (Table 5) [41], [45], [48]. We also used exon 3 of the single-copy nuclear gene *LEAFY* which is conserved across vascular plants, and there were near-universal primers available for the region (Table 5) [49]. Special care was taken to avoid contamination: a separate room was used for PCR amplification, and reactions were set under sterilised flow hoods using filter tips. Reactions were carried out in 25 µl volume containing 2 µl of template DNA, 1× Buffer, 1 mg/ml of Bovine Serum Albumin, 1.5 mM of MgCl2, 0.2 mM of each dNTP, 0.2–0.5 µM of each primer, and 1 U of DNA polymerase. The exception was *LEAFY* for which 3 mM of MgCl2 and 2.0 µM of each primer were used. PCR cycles had a 2 minute denaturation step at 94 or 95C depending on DNA polymerase enzyme used, 40 cycles of annealing with 94°C for 2 min, optimized annealing temperature for 1 min, and 72°C for 1 min, followed by 5 minute extension at 72°C. Annealing temperatures, optimized and tested using fresh test samples, were 45°C for *rbcL*, and 55°C for *LEAFY* and the P6 loop. Five µl of each PCR product was run on a 1.8% agarose gel with 0.5 ug/ml ethidium bromide, and all visible bands of the expected size were recorded as positive amplicons. Band presence on gel image was confirmed using the band autodetection option in GeneTools software (Syngene UK). No PCR products with double bands were seen. A fraction of samples (10–30% per PCR reaction) was sequenced in order to test for contamination. For these samples, PCR products were purified using exonuclease I and shrimp alkaline phosphatase (Exo/SAP) and sequenced using two reactions (forward and reverse) using Big Dye chemistry. After contig assembly using CodonCode Aligner (CodonCode Corporation) consensus sequences were BLAST-searched against the NCBI nucleotide database.

### Experimental Specimen Preparation

Eighteen species from 17 different families representing 18 orders were chosen for the experimental specimen study (Table S2). Five specimens were prepared from each plant, and each specimen was dried using a different specimen preparation technique to test the effect of drying techniques on DNA post-mortem damage. The five drying techniques included: (1) air drying at room temperature (20°C) over two weeks, (2) air drying using artificial heat (60°C) overnight, (3) alcohol drying over one day, (4) alcohol drying over two weeks, and (5) silica drying (leaves only). Air drying at room temperature was performed using a plant press with newspaper and blotting paper. Papers were exchanged every day, and the press was kept at room temperature until all plants were dry. Air drying with artificial heat was performed in an open cabinet drier in which heat is directed upwards through the plant press with newspaper and corrugates. Alcohol drying was performed by closing specimens between newspapers in an airtight plastic bag with 70% alcohol; specimens were left in alcohol for one day (method 3) and two weeks (method 4) before air drying them in artificial heat (60°C) in the open cabinet drier using fresh newspaper and corrugates. Once all specimens were dry, leaf fragments were weighed and 5 mg of each was used for DNA extraction following the DNeasy protocol.

## Supporting Information

File S1 **Details of DNA extraction protocols used.**
(DOC)

Table S1 **Details of the herbarium samples used in the study.**
(DOC)

Table S2 **Details of the experimentally dried herbarium samples.**
(DOC)

## References

[1] Medellín-Leal F (1975) Origenes, desarrollo historico y estado actual de los herbarios en el mundo. Bol Soc Bot Mex 1975 34: 3–26.

[2] Sheltler SG (1969) The herbarium: Past, present, and future. Proc Biol Soc Amer 82: 687–758.

[3] Holmgren PK, Holmgren NH, Barnett LC (1990) Index herbariorum. Part I: The herbaria of the world. New York: New York Botanical Garden. 693 p.

[4] Bebber DP, Carine MA, Wood JRI, Wortley AH, Harris DJ, et al (2010) Herbaria are a major frontier for species discovery. Proc Natl Acad Sci USA 107: 22169–22171. 21135225 10.1073/pnas.1011841108 PMC3009773

[5] Joppa LN, Roberts DL, Pimm SL (2011) How many species of flowering plants are there? Proc Biol Sci 278: 554–559. 20610425 10.1098/rspb.2010.1004 PMC3025670

[6] Staats M, Cuenca A, Richardson JE, Vrielink-van Ginkel R, Petersen G, et al (2011) DNA damage in plant herbarium tissue. PLoS ONE 6: e28448. 22163018 10.1371/journal.pone.0028448 PMC3230621

[7] Harris SA (1993) DNA analysis of tropical plant species: an assessment of different drying methods. P1ant Syst Evol 188: 57–64.

[8] Pyle MM, Adams RP (1989) In situ preservation of DNA in plant specimens. Taxon 38: 576–581.

[9] Erkens RHJ, Cross H, Maas JW, Hoenselaar K, Chatrou LW (2008) Assessment of age and greenness of herbarium specimens as predictors for successful extraction and amplification of DNA. Blumea 53: 407–428.

[10] Weising K, Nybom H, Wolff K, Meyer W (1995) DNA fingerprinting in plants and fungi. Boca Raton: CRC Press. p.322.

[11] Doyle JJ, Dickson EE (1987) Preservation of plant species for DNA restriction endonuclease analysis. Taxon 36: 715–722.

[12] Jobes DV, Hurley DL, Thien LB (1995) Plant DNA isolation: a method to efficiently remove polyphenolics, polysaccharides and RNA. Taxon 44: 379–386.

[13] Barnwell P, Blanchard AN, Bryant JA, Smirnoff N, Weir AF (1998) Isolation of DNA from the highly mucilagenous succulent plant *Sedum telephium* . Plant Mol Biol Rep 16: 133–138.

[14] de la Cruz M, Ramirez F, Hernandez H (1997) DNA isolation and amplification from cacti. Plant Mol Biol Rep 15: 319–325.

[15] Al-Shayji Y, Saleem M, Al-Amad S, Al-Awadhi S, Al-Salameen F (2004) Isolation and analysis of the total genomic DNA from the date palm (*P. dactylifera* L.) and related species. Acta Biotechnologica 14: 163–168.

[16] Fleischmann A, Heubl G (2009) Overcoming DNA extraction problems from carnivorous plants. An Jard Bot Madrid 66: 209–215.

[17] Michiels A, Van den Ende W, Tucker M, Van Riet L, Van Laere A (2003) Extraction of high-quality genomic DNA from latex-containing plants. Anal Biochem 315: 85–89. 12672415 10.1016/s0003-2697(02)00665-6

[18] Rogers SO, Bendich AJ (1985) Extraction of DNA from milligram amounts of fresh, herbarium and mummified plant tissues. Plant Mol Biol 5: 69–76. 24306565 10.1007/BF00020088

[19] Savolainen V, Cuénoud P, Spichiger R, Martinez MDP, Crèvecoeur M, et al (1995) The use of herbarium specimens in DNA phylogenetics: evaluation and improvement. Plant Syst Evol 197: 87–98.

[20] Drábková L, Kirschner J, Vleek C (2002) Comparison of seven DNA extraction and amplification protocols in historical herbarium specimens of Juncaceae. Plant Mol Biol Rep 20: 161–175.

[21] Jankowiak K, Buczkowska K, Szweykowska-Kulinska Z (2005) Successful extraction of DNA from 100-year-old herbarium specimens of the liverwort *Bazzania trilobata* . Taxon 54: 335–336.

[22] Li F-W, Kuo L-Y, Huang Y-M, Chiou W-L, Wang C-N (2010) Tissue-direct PCR, a rapid and extraction-free method for barcoding of ferns. Mol Ecol Res 10: 92–95. 10.1111/j.1755-0998.2009.02745.x 21564993

[23] Brockington SF, Mavrodiev E, Famdial J, Dhingra A, Soltis PS, et al (2008) Keep the DNA rolling: multiple displacement amplification of archival plant DNA extracts. Taxon 57: 944–948.

[24] Pan X, Urban AE, Palejev D, Schulz V, Grubert F, et al (2008) A procedure for highly specific, sensitive, and unbiased whole-genome amplification. Proc Natl Acad Sci USA 105: 15499–15504. 18832167 10.1073/pnas.0808028105 PMC2563063

[25] Ivanova NV, Fazekas AJ, Hebert PDN (2008) Semi-automated, membrane-based protocol for DNA isolation from plants. Plant Mol Biol Rep 26: 186–198.

[26] Doyle JJ, Doyle JL (1987) A rapid isolation procedure for small quantities of fresh leaf material. Phytochem Bull 19: 11–15.

[27] Höss M, Pääbo S (1993) DNA extraction from Pleistocene bones by a silica-based purification method. Nucl Acids Res 21: 3913–3914. 8396242 10.1093/nar/21.16.3913 PMC309938

[28] Hänni C, Brousseau T, Laudet V, Stehelin D (1995) Isopropanol precipitation removes PCR inhibitors from ancient bone extracts. Nucleic Acids Res 23: 881–882. 7708508 10.1093/nar/23.5.881 PMC306775

[29] Kalmar T, Bachrati CZ, Marcsik A, Rasko I (2000) A simple and efficient method for PCR amplifiable DNA extraction from ancient bones. Nucl Acids Res 28: E67. 10871390 10.1093/nar/28.12.e67 PMC102752

[30] Rohland N, Hofreiter M (2007b) Ancient DNA extraction from bones and teeth. Nature Protocols 2: 1756–1762. 17641642 10.1038/nprot.2007.247

[31] Soltis PS, Soltis DE (1993) Ancient DNA: prospects and limitations. New Zealand J Bot 31: 203–209.

[32] Zimmermann J, Hajibabaei M, Blackburn DC, Hanken J, Cantin E, et al. (2008) DNA damage in preserved specimens and tissue samples: a molecular assessment. Frontiers of Zoology 5: doi:10.1186/1742-9994-5-18. 10.1186/1742-9994-5-18 PMC2579423 18947416

[33] Telle S, Thines M (2008) Amplification of cox2 (∼620 bp) from 2 mg of up to 129 years old herbarium specimens, comparing 19 extraction methods and 15 polymerases. PLoS ONE 3: e3584. 18974835 10.1371/journal.pone.0003584 PMC2572190

[34] Rohland N, Hofreiter M (2007a) Comparison and optimization of ancient DNA extraction. BioTechniques 42: 343–352. 17390541 10.2144/000112383

[35] Pääbo S (1990) Amplifying ancient DNA. In: Innis MA, Gelfand DH, Sninsky JJ, White TJ, editors. PCR protocols: a guide to methods and applications. New York: Academic Press. 159–166.

[36] Schenck H (1887) Instrumente, Präparationsmethoden – Ueber die Schweinfurth’sche Methode, Pflanzen für Herbarien auf Reisen zu conserviren. Botanisches Centralblatt 35: 175–176.

[37] Schenck H (1888) Schweinfurth’s method for preserving plants. Bull Torrey Bot Club: 292–293.

[38] Schenck H (1889) Schweinfurth’s method for preserving plants. Kew Bull: 19–20.

[39] Taberlet P, Coissac E, Pompanon FO, Brochmann C, Willerslev E (2012) Towards next-generation biodiversity assessment using DNA metabarcoding. Mol Ecol 21: 2045–2050. 22486824 10.1111/j.1365-294X.2012.05470.x

[40] Hajibabaei M, Smith MA, Janzen DH, Rodriguez JJ, Whitfield JB, et al (2006) A minimalist barcode can identify a specimen whose DNA is degraded. Mol Ecol Notes 6: 959–964.

[41] CBOL Plant Working Group (2009) A DNA barcode for land plants. Proc Natl Acad Sci USA 106: 12794–12797. 19666622 10.1073/pnas.0905845106 PMC2722355

[42] Raye G, Miquel C, Coissac E, Redjadj C, Loison A, et al (2011) New insights on diet variability revealed by DNA barcoding and high-throughput pyrosequencing: chamois diet in autumn as a case study. Ecol Research 26: 265–276.

[43] Soininen EM, Valentini A, Coissac E, Miquel C, Gielly L, et al. (2009) Analysing diet of small herbivores: the efficiency of DNA barcoding coupled with high-throughput pyrosequencing for deciphering the composition of complex plant mixtures. Front Zoo 6: DOI:10.1186/1742-9994-6-16. 10.1186/1742-9994-6-16 PMC2736939 19695081

[44] Hofreiter M, HN Poinar, WG Spaulding, K Bauer, PS Martin, et al (2000) A molecular analysis of ground sloth diet through the last glaciation. Mol Ecol 9: 1975–1984. 11123610 10.1046/j.1365-294x.2000.01106.x

[45] Taberlet P, Coissac E, Pompanon F, Gielly L, Miquel C, et al (2007) Power and limitations of the chloroplast *trnL* (UAA) intron for plant DNA barcoding. Nucl Acids Res 35: e14. 17169982 10.1093/nar/gkl938 PMC1807943

[46] Metzker ML (2010) Sequencing technologies – the next generation. Nature Reviews Genetics 11: 31–46. 10.1038/nrg2626 19997069

[47] Thompson JF, Milos PM (2011) The properties and applications of single-molecule DNA sequencing. Genome Biol 12: 217. 21349208 10.1186/gb-2011-12-2-217 PMC3188791

[48] Kress WJ, Erickson DL (2007) A two-locus global DNA barcode for land plants: the coding *rbcL* gene complements the non-coding *trnH*-*psbA* spacer region. PLoS ONE 2: e508. 17551588 10.1371/journal.pone.0000508 PMC1876818

[49] Frohlich MW, Meyerowitz EM (1997) The search for flower homeotic gene homologs in basal angiosperms and gnetales: a potential new source of data on the evolutionary origin of flowers. Int J Plant Sci 158: S131–S142.

