# Supplementary material for: How to Open the Treasure Chest? Optimising DNA Extraction from Herbarium Specimens
Source: PLoS One. 2012 Aug 28;7(8):e43808. doi: 10.1371/journal.pone.0043808 (PMC3429509; doi:10.1371/journal.pone.0043808)
Supplement: File S1 — Details of DNA extraction protocols used. (DOC) [file pone.0043808.s001.doc]

**CTAB DNA EXTRACTION PROTOCOL**

1. Sample 5 mg of leaf tissue into tubes; add a pinch of sterilised sand into each tube to ease grinding of the tissue.
2. Preheat 1 ml 2X CTAB buffer per sample in a 65˚C water bath.
3. Grind samples using a Mixer Mill with metal beads. Perform short grinds (30 seconds to 1 minute) followed by cooling on ice to avoid heat damage to DNA. Alternatively, cool the grinding blocks in freezer prior to grinding. Stop once material is turned into a fine powder.
4. LYSIS: Add 1 ml of 2X CTAB buffer to each sample, and incubate at 65˚C for 60 minutes.
5. CHLOROFORM EXTRACTION: Add an equal volume (750µl) of SEVAG, mixing gently but thoroughly. Extract for up to one hour for slimy or mucilaginous samples by rocking on an orbital shaker.
6. Spin at 13,000 rpm (8,000 rpm for tubes larger than eppendorfs) for 10 minutes.
7. Remove aqueous (top) phase containing DNA and transfer to a new Eppendorf tube. Dispose of SEVAG and plant debris in the SEVAG waste container.
8. Repeat the chloroform extraction (stage 5-7).
9. PRECIPITATION: Add 2/3 volume of -20˚C isopropanol and mix gently to precipitate DNA. Leave in -20˚C freezer for up to two weeks.
10. WASH-UP: Spin in a centrifuge at 13,000 rpm for 10 minutes to collect precipitate. Pour off liquid in the waste container in the fume cupboard. Add 750 µl of ice-cold 70% ethanol, shake well to dislodge pellet to facilitate “washing”, and wash for 60 minutes.
11. Spin down DNA at 13,000 rpm for 5 minutes. Pour off liquid and drain upside down for 5-10 minutes to allow alcohol to evaporate. If some ethanol is still left in tubes after 5-10 mins, do an extra spin in centrifuge and pipette the ethanol out carefully. Drying for too long can cause the DNA to be difficult to dissolve. You can also dry the samples in vacuum centrifuge for c. 5 mins.
12. Resuspend your DNA in 100 µl of TE buffer (pH 8).
13. RNA’s REMOVAL: Add 1 µl (1 µl of RnaseA stock solution (Qiagen) per 100 µl DNA solution (100mg/ml)) per sample. Vortex, then incubate at 37˚C for 20 min.
14. PRECIPITATION: Add 100 µl of cold ammonium acetate (7.5M) and 750 µl of 100% alcohol per sample, and incubate on ice/freezer for 30 mins.
15. WASH-UP: Spin in a centrifuge at 13,000 rpm for 10 minutes to collect precipitate. Pour off liquid in the waste container in the fume cupboard. Add 500 µl of ice-cold 70% ethanol, shake well to dislodge pellets to facilitate “washing”, and wash for 30 minutes.
16. Spin down DNA at 13,000 rpm for 5 minutes. Pour off liquid and drain upside down for 5-10 minutes to allow alcohol to evaporate. If some ethanol is still left in tubes after 5-10 mins, do an extra spin in centrifuge and pipette the ethanol out carefully. Drying for too long can cause the DNA to be difficult to dissolve. You can also dry the samples in a vacuum centrifuge for c. 5 mins (low heat set).
17. Dissolve DNA in 75 µl of dH2O.

**RECIPES FOR CTAB EXTRACTION**

**2X CTAB buffer (250 ml)**

100mM Tris-HCl pH 8.0 25 ml (1M)
 1.4M NaCl 70 ml (5M)
 20mM EDTA 10 ml (0.5M)
 CTAB 5 g (2%) (hexadecyltrimethylammonium bromide)

Water until 250 ml

PVP-40 0.02g/1ml (polyvinylpyrrolidone)

**SEVAG**

24:1 chloroform : isoamyl alcohol

**Wash Buffer**

100% ETOH 75 ml (750 μl/sample)

7.5M NH_4_Ac 10 ml (100 μl/sample)

**TE buffer (100 ml at pH 8.0; 10mM Tris-HCl (pH 8) and 1mM EDTA) (1L/50ml)**

Tris-HCL (1M) 1 ml (10ml/500μl)

EDTA (100mM) 1 ml (2ml/100μl)

H2O 98 ml (1L/50ml)

**Ammonium acetate (NH4Ac) (50 ml) 7.5M (molecular weight = 77.08)**

ammonium acetate 28.905 g

ddH2O 50 ml

**UREA DNA EXTRACTION PROTOCOL**

1. Sample 5 mg of leaf tissue into tubes; add a pinch of sterilised sand into each tube to ease grinding of the tissue.
2. Grind samples using Mixer Mill with metal beads. Perform short grinds (30 seconds to 1 minute) followed by cooling on ice to avoid heat damage to DNA. Alternatively, cool the grinding blocks in freezer prior to grinding. Stop once material is turned into a fine powder.
3. Add 750 ul extraction buffer and incubate with slow mixing at 20 rpm for 2 weeks at room temperature.
4. Add 250 ul of 3M potassium acetate.
5. Incubate on ice for 10 minutes.
6. Centrifuge 5 minutes at maximum speed. Transfer lysate to new eppendorf.
7. Add an equal volume (750µl) of SEVAG, mixing gently but thoroughly. Extract for up to one hour for slimy or mucilaginous samples by rocking on an orbital shaker.
8. Spin at 13,000 rpm (8,000 rpm for tubes larger than eppendorfs) for 10 minutes.
9. Remove aqueous (top) phase containing DNA and transfer to a new Eppendorf tube. Dispose of SEVAG and plant debris in the SEVAG waste container.
10. Repeat the chloroform extraction (stage 5-7).
11. Add 2/3 volume of -20˚C isopropanol and mix gently to precipitate DNA. Leave in -20˚C freezer for 60 minutes.
12. Spin in a centrifuge at 13,000 rpm for 10 minutes to collect precipitate. Pour off liquid in the waste container in the fume cupboard.
13. Resuspend pellet in 400 ul AP1 buffer (DNEasy kit) with 4 ul RNase A. Follow DNeasy Plant Miniprep protocol from here on.

**RECIPES FOR UREA EXTRACTION PROTOCOL**

**Extraction buffer (10 ml)**

7 M urea 4.2 g

0.3 M NaCl (5 M stock) 600 µl

20 mM EDTA pH 8.0 (o.5 M stock) 400 µl

50 mM Tris-HCl, pH 8.0 (1 M stock) 500 µl

1% (w/v) SDS (sodium dodecyl sulphate) 0.1g

**Potassium acetate (100 ml)**

Potassium acetate 29.4 g

H2O 30 ml

Adjust pH to 4.7 with Acetic acid (glacial)

Store at 4C
