# Supplementary material for: How to Open the Treasure Chest? Optimising DNA Extraction from Herbarium Specimens
Source: PLoS One. 2012 Aug 28;7(8):e43808. doi: 10.1371/journal.pone.0043808 (PMC3429509; doi:10.1371/journal.pone.0043808)
Supplement: Table S1 — Details of the herbarium samples used in the study. (DOC) [file pone.0043808.s002.doc]

Table S1 Details of the herbarium samples used in the study. All samples are deposited at the Royal Botanic Garden Edinburgh (E).

|  | **Order** | **Family** | **Plant species** | **Collector and collectors #** | **Age** | **Country** | **Likely drying method** | **Known issues** | **Leaf chemistry** | **Reference** |
| --- | --- | --- | --- | --- | --- | --- | --- | --- | --- | --- |
| 1 | Acorales | Acoraceae | *Acorus calamus* | Sinclair 10149 | 54 | U.K. | A | SA |  | Marongiu et al. 2005; Kumar et al. 2000 |
| 2 | Alismatales | Zosteraceae | *Zostera marina* | Sinclair 500 | 85 | U.K. | A | MU | PP, PS | Ovodava et al. 1968; Alemzadeh et al. 2005; |
| 3 | Apiales | Apiaceae | *Foeniculum vulgare* | Sinclair 7095 | 60 | U.K. | A |  | PP | Kaur & Arora 2009 |
| 4 | Aquifoliales | Aquifoliaceae | *Ilex aquifolium* | Heywood & Davis 268 | 64 | Spain | A | TO | PP | Ricco et al. 1991 |
| 5 | Arecales | Arecaceae | *Areca catechu* | Sinclair 3272 | 69 | India | A or L | TO | PP | Wang et al. 1997 |
| 6 | Asparagales | Orchidaceae | *Habenaria repens* | Tharp s.n. | 76 | U.S.A. | A |  | PP, PS | Johnson et al. 1999 |
| 7 | Asterales | Asteraceae | *Cichorium intybus* | Lonsley s.n. | 58 | U.K. | A | SA | PP, PS | Rossetto et al. 2005; Gupta & Ansari 2005 |
| 8 | Austrobaileyales | Schisandraceae | *Kadsura heteroclita* | Tsang 25239 | 77 | China | L |  | PP | Pu et al. 2007 |
| 9 | Brassicales | Brassicaceae | *Arabidopsis thaliana* | CONTROL | - | U.K. | S |  |  |  |
| 10 | Brassicales | Brassicaceae | *Arabidopsis thaliana* | Anthony 1798 | 54 | U.K. | A |  |  |  |
| 11 | Buxales | Buxaceae | *Sarcococca hookeriana* | Hu 8357 | 66 | China | A |  |  |  |
| 12 | Caryophyllales | Chenopodiaceae | *Chenopodium album* | Lonsley s.n. | 64 | U.K. | A |  | OX | Nuss & Loewus 1978 |
| 13 | Celastrales | Celastraceae | *Maytenus ilicifolia* | Luis 20030 | 62 | Brazil | A or L |  | PP, PS | Cipriani et al. 2009; Marino et al. 2009; |
| 14 | Commelinales | Commelinaceae | *Tradescantia* sp. | Ahles & Duke 48013 | 54 | U.S.A. | A |  |  |  |
| 15 | Crossosomatales | Stachyuraceae | *Stachyurus himalaicus* | Forrest 25165 | 88 | China | A or L |  |  |  |
| 16 | Cucurbitales | Begoniaceae | *Begonia urticae* | Balls 7503 | 74 | Colombia | A | SU | OX | Crombie 1954 |
| 17 | Dioscoreales | Dioscoreaceae | *Dioscorea zingiberensis* | Yu 14132 | 75 | China | A |  | PP | Qin et al. 2009 |
| 18 | Dipsacales | Adoxaceae | *Viburnum tinus* | Mello s.n. | 63 | U.S.A. | A |  | PP | Mohamed et al. 2005 |
| 19 | Ericales | Sapotaceae | *Manilkara hexandra* | Raizada 21228 | 59 | India | A | SA |  |  |
| 20 | Fabales | Leguminosae | *Inga striata* | Duarle 4760 | 53 | Brazil | A |  | PP |  |
| 21 | Fagales | Juglandaceae | *Juglans regia* | Koelz 11656 | 75 | Afganistan | A |  | PP | Pereira et al. 2007 |
| 22 | Gentianales | Rubiaceae | *Uncaria ferrea* | Sinclair & Kiah 38818 | 63 | Singapore | L |  | PS | Heitzman et al. 2005 |
| 23 | Geraniales | Geraniaceae | *Pelargonium cortusifolium* | Dunter 1363 | 83 | South Africa | A |  | EO, PP | Williams et al. 1997 |
| 24 | Ginkgoales | Ginkgoaceae | *Gingko biloba* | Chiao & Fan 547 | 74 | China | A |  | PP | van Beek 2002 |
| 25 | Lamiales | Phrymaceae | *Rehmannia glutinosa* | Jacot s.n. | 83 | China | A or L |  |  |  |
| 26 | Laurales | Lauraceae | *Cinnamomum burmannii* | Chun 5943 | 92 | China | A |  | PP, PS | Shan et al. 2007 |
| 27 | Liliales | Liliaceae | *Lilium speciosum* | Steward 2662 | 90 | China | A |  |  |  |
| 28 | Magnoliales | Annonaceae | *Annona squamosa* | Stainton, Sykes & Williams 5262 | 58 | Nepal | A |  | PP, PS | Chaves 1997; Saluja & Santani 1990; |
| 29 | Malpighiales | Phyllanthaceae | *Phyllanthus maderaspatensis* | Sinclair 3563 | 68 | India | A | SA | PP | Komuraiah et al. 2009 |
| 30 | Malvales | Dipterocarpaceae | *Dipterocarpus baudii* | Sinclair 7842 | 59 | Malaysia | L |  | PP | Joshi 2003 |
| 31 | Myrtales | Onagraceae | *Epilobium hirsutum* | Dunk, Halliday, & Mehlein 157 | 54 | U.K. | A | SA | PP | Barakat et al. 1997 |
| 32 | Nymphaeales | Nymphaeaceae | *Nuphar lutea* | Katanskaya s.n. (E) | 54 | Russia | A |  | SU |  |
| 33 | Oxalidales | Oxalidaceae | *Oxalis acetosella* | Sinclair 791 | 79 | U.K. | A |  | OX |  |
| 34 | Piperales | Piperaceae | *Piper nigrum* | Koelz 11206 | 75 | India | L |  | EO, PP | Parmar et al. 1998 |
| 35 | Poales | Poaceae | *Oryza sativa* | Haine s.n. | 52 | Iraq | A |  |  |  |
| 36 | Poales | Poaceae | *Sorghum bicolor* | Lau 5371 | 77 | China | A |  | PP | Awika & Rooney 2004 |
| 37 | Proteales | Proteaceae | *Protea neriifolia* | Rodin 1060 | 65 | South Africa | A |  | PP | Verotta et al. 1999 |
| 38 | Ranunculales | Berberidaceae | *Berberis angulosa* | Stainton, Sykes & Williams 9109 | 58 | Nepal | A | TO | PP | Peng et al. 2006 |
| 39 | Rosales | Crassulaceae | *Sedum telephium* | Sinclair 4752 | 65 | U.K. | A | SU | MA | Conti & Smirnoff 1994 |
| 40 | Santalales | Santalaceae | *Santalum austrocaledonium* | McKee 1983 | 57 | New Caledonia | A |  | EO, PP | Bottin et al. 2007 |
| 41 | Sapindales | Rutaceae | *Citrus aurantium* | Meyer & Rogers 2837 | 64 | Mexico | L |  | CA, EO, PP | He et al. 1997 |
| 42 | Sapindales | Meliaceae | *Aglaia rubiginosa* | Sinclair 8060 | 58 | Singapore | L |  |  |  |
| 43 | Saxifragales | Grossulariaceae | *Ribes aureum* | Balls 8349 | 81 | U.S.A. | A |  | PP | Mexal & Martin 1977 |
| 44 | Solanales | Solanaceae | *Solanum nigrum* | Polunin 1858 | 63 | Nepal | A |  | PP | Shivakumar et al. 2009 |
| 45 | Trochodendrales | Trochodendraceae | *Tetracentron sinense* | Yu 19217 | 74 | China | A |  |  |  |
| 46 | Unplaced | Dilleniaceae | *Curatella americana* | Steinbach 7316 | 87 | Bolivia | A or L |  | PP | El-Azizi et al. 1980 |
| 47 | Vitales | Vitaceae | *Vitis vinifera* | Davis 19572 | 60 | Turkey | A or L |  | PP | Patil et al. 1998 |
| 48 | Zingiberales | Zingiberaceae | *Alpinia galanga* | Sinclair 5035 | 64 | Singapore | L |  | EO | Jirovetz et al. 2003 |

**Abbreviations used**

A = air dried

CA = high in citric acid

EO = presence of essential oils

L = alcohol treated before drying

MA = high in malic acid

MU = mushy leaf texture

OX = high in oxalic acid

PP = high in polyphenols

PS = high in polysaccharides

S = silica dried

SA = contains sap or resin

SU = succulent leaves

TO = tough leaf texture

**References**

Alemzadeh A, Fujie M, Usami S, Yamada T (2005) Isolation of high-quality RNA from high-phenolic tissues of eelgrass (*Zostera marina* L.) by keeping temperature low. Plant Mol Biol Rep 23: 421a-421h.

Awika JM, Rooney LW (2004) *Sorghum* phytochemicals and their potential impact on human health. Phytochemistry 65: 1199–1221.

Barakat HH, Hussein SAM, Marzouk MS, Merfort I, Linscheid M, Nawwar MAM (1997). Polyphenolic metabolites of *Epilobium hirsutum*. Phytochemistry 46: 935-941.

Bottin L, Isnard C, Lagrange A, Bouvet JM (2007) Comparative molecular and phytochemical study of the tree species *Santalum austrocaledonicum* (Santalaceae) distributed in the New-Caledonian archipelago. Chem Biodivers 4: 1541-1556.

Chaves MH (1997) Analysis of extracts of plants by TLC: A methodology applied in the “organic chemistry” discipline. Quím Nova 20: 560-562.

Cipriani TR, Mellinger CG, de Souza LM, Baggio CH, Freitas CS, Marques MCA, Gorin PJA, Sassaki GL, Iacomini M (2009) Polygalacturonic acid: Another anti-ulcer polysaccharide from the medicinal plant *Maytenus ilicifolia*. Carbohydrate Polymers 78: 361–363.

Conti S, Smirnoff N (1994) Rapid triggering of malate accumulation in the C3/CAM intermediate plant *Sedum telephium*: relationship with water status and phosphoenolpyruvate carboxylase. J Exp Bot 45: 1613-1621.

Crombie WML (1954) Oxalic acid metabolism in *Begonia semperflorens*. J Exp Bot 5: 173-183.

El-Azizi MM, Ateya AM, Svoboda GH, Schiff PL, Slatkin DJ, Knapp JE (1980) Chemical constituents of *Curatella americana* (Dilleniaceae). J Pharm Sci 69: 360-361.

Gupta SK, Sansari SH (2005) Review on phytochemical and pharmacological aspects of *Cichorium itybus* Linn. Asian J Chem 17: 33-36.

He X-G, Lian L-Z, Lin L-Z, Bernart MW (1997) High-performance liquid chromatography–electrospray mass spectrometry in phytochemical analysis of sour orange (*Citrus aurantium* L.). J Chromatogr A, 791: 127–134.

Heitzman ME, Neto CC, Winiarz E, Vaisberg AJ, Hammond GB (2005) Ethnobotany, phytochemistry and pharmacology of *Uncaria* (Rubiaceae). Phytochemistry 66: 5-29.

Johnson MK, Alexander KE, Lindquist N, Loo G (1999) A phenolic antioxidant from the freshwater orchid, *Habenaria repens*. Comp Biochem & Phys C 122: 211–214.

Joshi K (2003) Leaf flavonoid patterns in *Dipterocarpus* and *Hopea* (Dipterocarpaceae). Bot J Linn Soc 143: 43–46.

Kaur GJ, Arora DS (2009) Antibacterial and phytochemical screening of *Anethum graveolens*, *Foeniculum vulgare* and *Trachyspermum ammi*. BMC Compl Alternative Med 9: 30.

Komuraiah A, Bolla K, Rao KN, Ragan A, Rajul VS, Charya MAS (2009) Antibacterial studies and phytochemical constituents of South Indian *Phyllanthus* species. African J Biotechn 8: 4991-4995.

Kumar VS, Srivastava RK, Krishna A, Tomar VKS, Singh AK, Kumar S (2000) Cultivation, chemistry, biology and utilization of bach (*Acorus calamus*): A review. J Med Aromat Plant Sci 22: 338-348.

Marino DC, Sabino LZL, Armando, J, Ruggiero A, Moya HD (2009) Analysis of the polyphenols content in medicinal plants based on the reduction of Cu(II)/Bicinchoninic complexes. Agric Food Chem 57: 11061–11066.

Marongiu B, Piras A, Porcedda S, Scorciapino A (2005) Chemical composition of the essential oil and supercritical CO2 extract of *Commiphora myrrha* (Nees) Engl. and of *Acorus calamus* L. J Agric Food Chem 53: 793-794.

Mexal J, Martin WC (1977) Chemotaxonomy of *Ribes*. The Southwest Naturalist 21: 523-530.

Mohamed MA, Marzouk MSA, Moharram FA, El-Sayed MM, Baiuomy AR (2005) Phytochemical constituents and hepatoprotective activity of *Viburnum tinus* Phytochemistry 66: 2780–2786.

Nuss RF, Loewu FA (1978) Further studies on oxalic acid biosynthesis in oxalate accumulating plants. Plant Phys 61: 590-592.

Ovodova RG, Vaskovsky VE, Ovodov YS (1968) The pectic substances of Zosferaceae. Carbohyd Res 6: 1328-332.

Parmar VS, Jain SC, Gupta S, Talwar S, Rajwanshi VK, Kumar R, Azim A, Malhotra S, Kumar N, Jain R, Sharma NK, Tyagi OD, Lawrie SJ, Errington W, Howarth OW, Olsen CE, Singh SK, Wengel J (1998). Polyphenols and alkaloids from *Piper* species. Phytochemistry 49: 1069-1078.

Patil, SG, Honrao BK, Karkamkar SP (1998) Phyto-chemical studies in the genus *Vitis* L. (fam. Vitaceae) and their significance. J Econ Tax Bot 22: 371-375.

Peng Y, Chen S-B, Liu Y, Chen S-L, Xiao P-G (2006) A pharmacophylogenetic study of the Berberidaceae (s.l.). Acta Phytotax Sin 44: 241-257.

Pereira JA, Oliveira I, Sousa A, Valentão P, Andrade PB, Ferreira ICFR, Ferreres F, Bento A, Seabra R, Estevinho L (2007) Walnut (*Juglans regia* L.) leaves: Phenolic compounds, antibacterial activity and antioxidant potential of different cultivars. Food Chem Toxicol 45: 2287–2295.

Pu J-X, Yang L-M, Xiao W-L, Li R-T, Lei C, Gao X-M, Huang S-X, Li S-H, Zheng Y-T, Huang H, Sun H-D (2008) Compounds from *Kadsura heteroclita* and related anti-HIV activity. Phytochemistry 69: 1266–1272.

Qin Y, Qu X, Huang W, Gong G, Li D, He Y, Zhao Y (2009) Acute toxicity and sub-chronic toxicity of steroidal saponins from *Dioscorea zingiberensis* C.H.Wright in rodents. J Ethnopharm 126: 543–550

Ricco RA, Wagner ML, Gurni AA (1991) Comparative study of flavonoids on six austral South American species of the genus *Ilex*. Acta Farm Boinaerense 10: 29-36.

Rossetto M, Lante A, Vanzani P, Spettoli P, Scarpa M, Rigo A (2005) Red chicories as potent scavengers of highly reactive radicals: A study on their phenolic composition and peroxyl radical trapping capacity and efficiency. J Agric Food Chem 53: 816-817.

Saluja AK, Santani DD (1990) Phytochemical study of *Annona squamosa*. Fitoterapia 61: 359-360.

Shan B, Cai Y-Z, Brooks JD, Corke H (2007) Antibacterial properties and major bioactive components of cinnamon stick (*Cinnamomum burmannii*): Activity against foodborne pathogenic bacteria. J Agric Food Chem 55: 548-549.

Shivakumar SL, Asma S, Karigar CS (2009) Phytochemical distribution profile among Solanaceae members. Vegetos 22: 59-66.

*Uncaria* (Rubiaceae). Phytochemistry 66: 5–29.

van Beek TA (2002) Chemical analysis of *Ginkgo biloba* leaves and extracts. J Chromatogr A 967: 21–55.

Verotta L, Orsini F, Pelizzoni F, Torri G, Rogers CB (1999) Polyphenolic glycosides from African Proteaceae. J Nat Prod 62: 1526-1531.

Wang C-K, Lee W-H, Peng C-H (1997) Contents of phenolics and alkaloids in *Areca catechu* Linn. during maturation. J Agric Food Chem 45: 1185-1188.

Williams CA, Harborne JF, Newman M, Greenham J, Eagles J (1997) Chrysin and other leaf exudate flavonoids in the genus *Pelargonium*. Phytochemistry 46: 1349-1353.
