# Supplementary material for: How to Open the Treasure Chest? Optimising DNA Extraction from Herbarium Specimens
Source: PLoS One. 2012 Aug 28;7(8):e43808. doi: 10.1371/journal.pone.0043808 (PMC3429509; doi:10.1371/journal.pone.0043808)
Supplement: Table S2 — Details of the experimentally dried herbarium samples. (DOC) [file pone.0043808.s003.doc]

**Table S2** Details of the experimentally dried herbarium samples.

| **#** | **Order** | **Family** | **Plant species** | **Accession Number at the Royal Botanic Garden Edinburgh** |
| --- | --- | --- | --- | --- |
| **1** | Aquifoliales | Aquifoliaceae | *Ilex aquifolium* L. | 19784139 |
| **2** | Arecales | Arecaceae | *Areca* sp. | 19973669 |
| **3** | Brassicales | Brassicaceae | *Arabidopsis thaliana* (L.) Heynh. | Weed in the garden outside |
| **4** | Commelinales | Commelinaceae | *Commelina communis* L. | Weed in the glasshouses |
| **5** | Cucurbitales | Begoniaceae | *Begonia dietrichiana* Irmsch. | 20030638A |
| **6** | Ericales | Sapotaceae | *Manilkara zapota* (L.) P. Royen | 9340163 |
| **7** | Fabales | Leguminosae | *Inga spectabilis* (Vahl) Willd. | 19990573 |
| **8** | Fagales | Juglandaceae | *Juglans ailanthifolia* Carrière | 19687699A |
| **9** | Geraniales | Geraniaceae | *Pelargonium sublignosum* R. Knuth | 20001186A |
| **10** | Ginkgoales | Ginkgoaceae | *Ginkgo biloba* L. | 19687595B |
| **11** | Lamiales | Acanthaceae | *Acanthus dioscoridis* L. | 19890362A |
| **12** | Oxalidales | Oxalidaceae | *Oxalis acetosella* L. | 20030016A |
| **13** | Poales | Poaceae | *Oryza sativa* L. | 19701631 |
| **14** | Ranunculales | Berberidaceae | *Berberis angulosa* Wall. ex Hook. f. & Thoms. | 20011097H |
| **15** | Ranunculales | Ranunculaceae | *Clematis* sp. | 19763021G |
| **16** | Saxifragales | Crassulaceae | *Sedum telephium* L. | 19744302 |
| **17** | Solanales | Solanaceae | *Solanum dulcamara* L. | Weed in the garden outside |
| **18** | Zingiberales | Zingiberaceae | *Alpinia galanga* (L.) Willd. | 19771077B |
